# P-978. Effect of the implementation of an antimicrobial stewardship program in the intensive care unit of a teaching hospital in Nicaragua

**DOI:** 10.1093/ofid/ofaf695.1177

**Published:** 2026-01-11

**Authors:** Irene Díaz-Blanco, Roger Maliaños-Miranda, Guillermo D Porras-Cortés

**Affiliations:** Hospital Dr. Fernando Vélez Paiz, Managua, Managua, Nicaragua; Hospital Dr. Fernando Vélez Paiz, Managua, Managua, Nicaragua; Hospital Dr. Fernando Vélez Paiz, Managua, Managua, Nicaragua

## Abstract

**Background:**

Antimicrobial resistance is a public health problem, with a significant impact on patient survival as well as economic and social repercussions. The antimicrobial stewardship (AMS) program is a strategy to mitigate the problem or bacterial resistance. The objective of this study was to evaluate the effect of the implementation of an AMS program model in the ICU of a teaching hospital in Nicaragua.

**Table 1.**
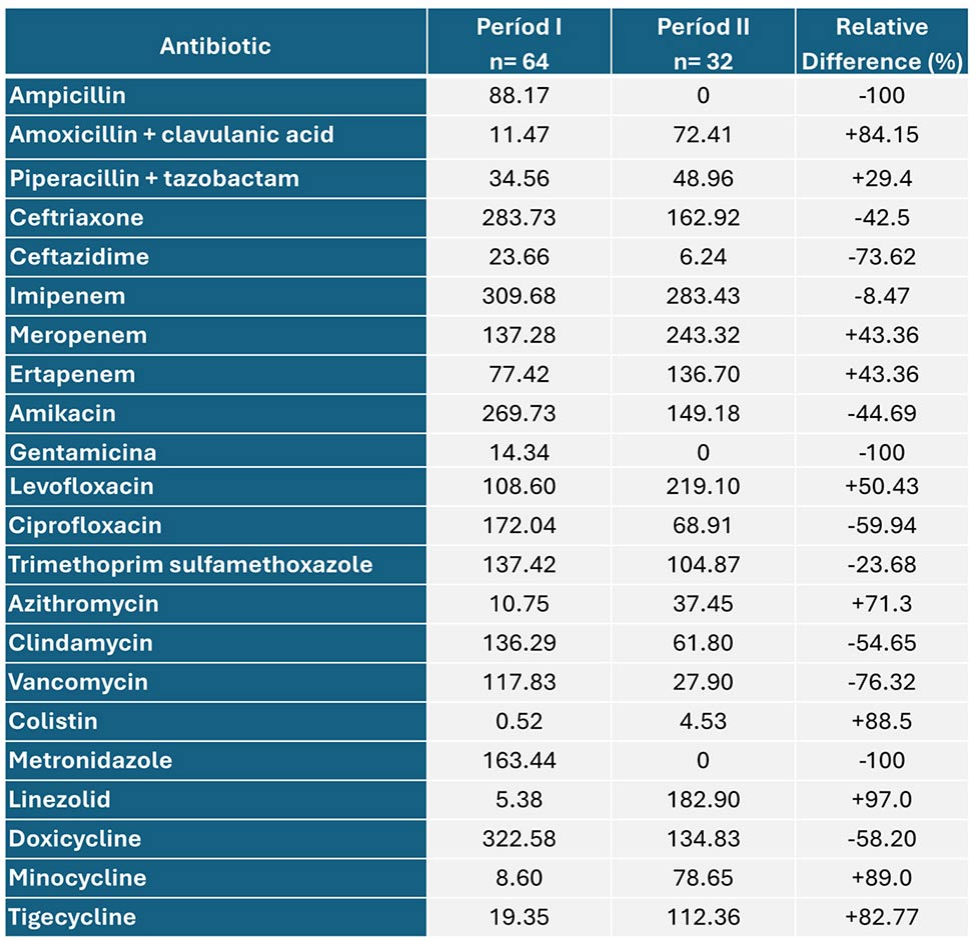
Comparison of use of antibiotics between period I and period II measured as DDD/1,000 patients-day in ICU at Hospital Dr. Fernando Vélez Paiz in Nicaragua

**Table 2.**
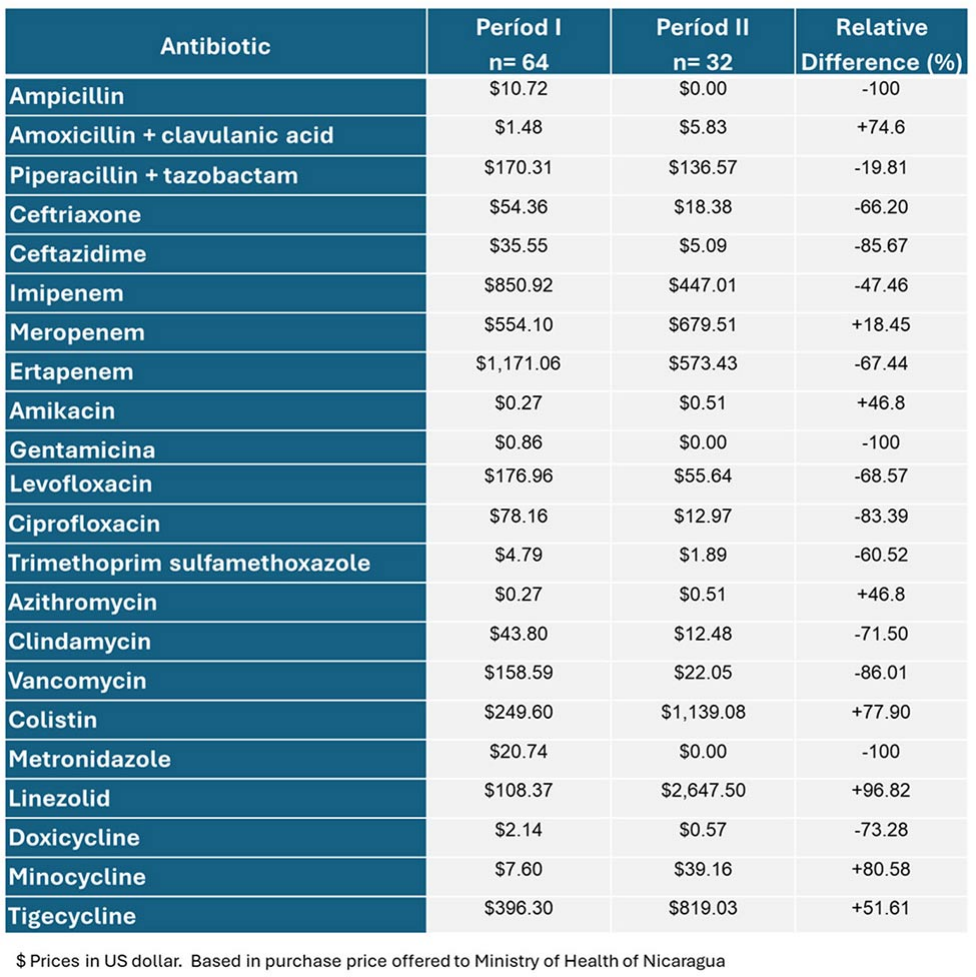
Expenses in US dollars in antibiotics in period I and II in ICU in Hospital Dr. Fernando Vélez Paiz, Nicaragua.

**Methods:**

This is an analytical, observational, cohort, ambispective study; performed between July 2022 and June 2023 in the Dr. Fernando Vélez Paiz Hospital in Managua, Nicaragua. A total of 96 patients were included (64 patients in the period I or pre-intervention and 32 patients in the period II or intervention). The intervention was the implementation of a systematic AMS program in the ICU whose activities were daily morning clinical round by the infectious diseases staff to make initial therapeutic decisions and follow-up, subsequently microbiological round in the laboratory to review of cultures and to proceed with the respective treatment adjustments. In the afternoon, night and weekends, antimicrobials were approved or exchanged by the staff shift on site or by telemedicine. Antibiotic consumption per defined daily dose (DDD/1000 patient days), antibiotic expenditure in US dollars, and mortality rate in each period were analyzed.

**Table 3.**
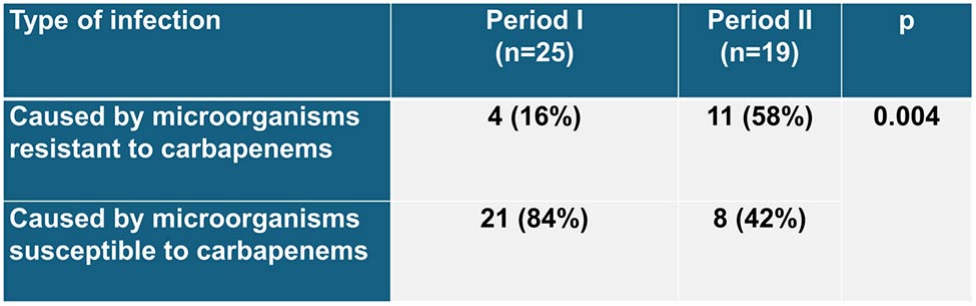
Infections by carbapenem resistant bacteria in period I and II in ICU of the Hospital Dr. Fernando Vélez Paiz, Nicaragua

**Results:**

In period I, the most frequent diagnosis was urinary tract infection, and in period II, intra-abdominal infection. A decrease in antibiotic consumption measured by DDD was observed in 12 of 22 antimicrobials (54.4%) in the period II. Among the antimicrobials that registered a decrease in consumption were: vancomycin, ceftazidime and ciprofloxacin with a reduction of 76.32%, 73.62% and 59.94% respectively (Table 1). Spending in US dollars decreased in 15 out of 22 antimicrobials (68.2%) (Table 2). In the AMS program period, there was an increase in the consumption of colistin, minocycline and tigecycline, explained by an increase in carbapenem-resistant Enterobacterales infections most of them expresing NDM metalobetalactamase (Table 3).

**Conclusion:**

AMS program showed a reduction in DDD in more than half of the antibiotics and also a reduction in costs of some antibiotics. An improving effect in the clinical outcomes was not demonstrated.

**Disclosures:**

All Authors: No reported disclosures

